# AZU1 as a DNA Methylation-Driven Gene: Promoting Oxidative Stress in High-Altitude Pulmonary Edema

**DOI:** 10.3390/antiox14070835

**Published:** 2025-07-08

**Authors:** Qiong Li, Zhichao Xu, Qianhui Gong, Liyang Chen, Xiaobing Shen, Xiaowei Chen

**Affiliations:** 1 School of Nursing, Nanjing University of Chinese Medicine, Nanjing 210023, China; liqiong@njucm.edu.cn; 2 Key Laboratory of Environmental Medicine Engineering, Ministry of Education, School of Public Health, Southeast University, Nanjing 210018, China; 220213998@seu.edu.cn (Z.X.); 220223631@seu.edu.cn (Q.G.); 230239639@seu.edu.cn (L.C.); xb.shen@seu.edu.cn (X.S.); 3 School of Elderly Care Services and Management, Nanjing University of Chinese Medicine, Nanjing 210023, China

**Keywords:** 850K DNA methylation chips, RRBS, TBS, DNA methylation, AZU1, high-altitude pulmonary edema, HUVEC

## Abstract

High-altitude pulmonary edema (HAPE) is a severe condition associated with high-altitude environments, and its molecular mechanism has not been fully elucidated. This study systematically analyzed the DNA methylation status of HAPE patients and healthy controls using reduced-representation bisulfite sequencing (RRBS) and 850K DNA methylation chips, identifying key differentially methylated regions (DMRs). Targeted bisulfite sequencing (TBS) revealed significant abnormalities in DMRs of five genes, azurocidin 1 (AZU1), growth factor receptor bound protein 7 (GRB7), mannose receptor C-type 2 (MRC2), RUNX family transcription factor 3 (RUNX3), and septin 9 (SEPT9). The abnormal expression of AZU1 was validated using peripheral blood leukocytes from HAPE patients and normal controls, as well as rat lung tissue, indicating its potential importance in the pathogenesis of HAPE. To further validate the function of AZU1, we conducted experimental studies using a hypobaric hypoxia injury model in Human Umbilical Vein Endothelial Cells (HUVEC). The results showed that AZU1 was significantly upregulated under hypobaric hypoxia. Knocking down AZU1 mitigates the reduction in HUVEC proliferation, angiogenesis, and oxidative stress damage induced by acute hypobaric hypoxia. AZU1 induces cellular oxidative stress via the p38/mitogen-activated protein kinase (p38/MAPK) signaling pathway. This study is the first to elucidate the mechanism of AZU1 in HAPE via the p38/MAPK pathway, offering novel insights into the molecular pathology of HAPE and laying a foundation for future diagnostic and therapeutic strategies.

## 1. Introduction

Individuals who have resided at low altitudes for an extended period are at an elevated risk of developing high-altitude pulmonary edema (HAPE) upon their initial ascent to high altitudes. The estimated incidence rate of HAPE ranges from approximately 0.2% to 4% [1]. Without adequate medical care, the mortality rate can rise to as high as 40% to 50% [2]. The occurrence of HAPE is closely correlated with pulmonary function, influenced significantly by both environmental and genetic factors. Epigenetic DNA modifications, which reflect genetic predisposition and environmental exposure throughout an individual’s life, can help identify genes that impact lung function [3]. DNA methylation is currently the most extensively studied epigenetic modification, facilitated by high-throughput, reproducible platforms that offer genome-wide coverage. DNA methylation refers to the covalent bond formed between the 5′ carbon atom of a cytosine nucleotide in a CpG dinucleotide (5′-cytosine-phosphate-guanine-3′) and a methyl group, catalyzed by DNA methyltransferase, resulting in 5-methylcytosine [4]. DNA hypermethylation can directly inhibit transcription or indirectly repress gene expression through transcriptional silencing, whereas DNA hypomethylation can activate transcription and enhance gene expression [5]. However, the dynamic regulatory mechanisms of genome-wide DNA methylation in the development of HAPE remain largely unknown.

The methylation status of genomes in various samples (tissues, cells, individuals, etc.) can vary, with differentially methylated regions (DMRs) playing a role in the transcriptional regulation of genes. DMRs between Tibetans and non-Tibetans were enriched around high-altitude adaptation genes, such as endothelial PAS domain protein 1 (EPAS1) and egl-9 family hypoxia inducible factor 1 (EGLN1), suggesting that alterations in DNA methylation play a crucial role in high-altitude adaptation [6]. Furthermore, differential methylation in EGLN1 is associated with blood oxygen saturation and plasma protein levels, as observed in cases of HAPE [7]. Von Hippel-Lindau (VHL) methylation has been shown to upregulate the hypoxia-inducible factor-1α (HIF-1α)/erythropoietin (EPO) pathway in rat bone marrow, leading to excessive red blood cell proliferation in chronic mountain sickness [8]. A subsequent study indicated that genetic differences and abnormal methylation in the apelin system can predict the risk of HAPE [9].

While some studies have investigated the methylation patterns associated with high-altitude adaptation and acclimatization, the identification of DNA methylation driving genes in HAPE remains limited. We hypothesize that changes in DNA methylation of genes play a crucial role in the development of HAPE. To test this hypothesis, we performed DMR analysis to compare differences between HAPE and controls. Therefore, in the first stage, we performed reduced-representation bisulfite sequencing (RRBS) on peripheral blood samples. In the second stage, we validated the RRBS results using 850k methylation sequencing. In the third stage, we further validated these findings with a larger sample size, using targeted bisulfite sequencing (TBS), leading to the identification of key DMR genes. In the fourth stage, we conducted wet lab experiments to validate a significant gene and investigate its role in the occurrence and development of HAPE, focusing on its mechanism of action in vascular endothelial cells.

## 2. Materials and Methods

### 2.1. Subjects

**Figure 1:**
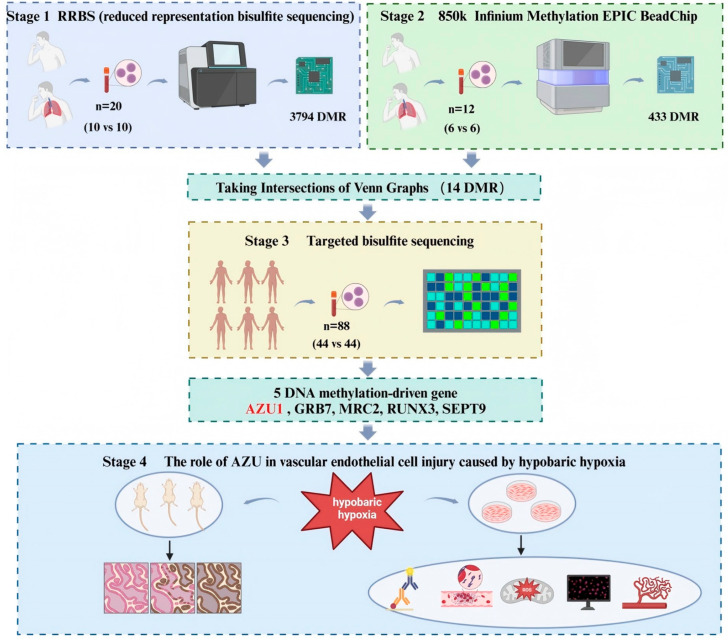
The flowchart of this study.

Participants for this study were recruited from Houbei Hospital in Lhasa, Tibet. Recruitment occurred between February 2021 and July 2023. Additional control samples were obtained from a cohort of healthy volunteers who traveled to Tibet specifically for this study. All participants and healthy volunteers were Han Chinese, ranging from 18 to 65 years. The diagnosis of HAPE was established by a team of expert physicians and radiologists through a comprehensive assessment of clinical data. Key symptoms included dyspnea; cyanosis; dry cough upon exertion; pink, foamy sputum; orthopnea; and fever. Additionally, chest X-ray findings were utilized to inform the diagnosis. Exclusion criteria for the study included (1) long-term residence in high-altitude areas; (2) serious physical illnesses, including neurogenic, endocrine, metabolic, autoimmune, cardiovascular, and cerebrovascular diseases; (3) non-Han Chinese ancestry within three generations; and (4) any other reasons that rendered individuals unsuitable or unable to complete the study. Ethical approval for the study was obtained from the XIZANG Center for Disease Control and Prevention Ethics Review Committee (reference number 2023-001). Prior to the commencement of the study, written informed consent was obtained from all participants. The study’s flowchart is presented in Figure 1.

### 2.2. Whole-Blood DNA Extraction

HAPE patients provided 5 mL of anticoagulated blood samples on the first day of hospitalization, prior to any treatment interventions. Healthy volunteers collected 5 mL of blood samples on the third day after entering high-altitude areas, placing them into anticoagulant tubes for DNA methylation sequencing. Once collected, the blood samples were stored at −80 °C for future use. DNA was extracted from the blood samples using the Blood Genome DNA Extraction Kit (Concert, Co. Ltd., Xiamen, China). The concentration and purity of the extracted DNA were quantified using a NanoDrop 2000c spectrophotometer (Thermo Fisher Scientific, Waltham, MA, USA).

### 2.3. Reduced-Representation Bisulfite Sequencing (RRBS)

Initially, blood samples from 10 HAPE cases and 10 sex- and age-matched control cases were selected for RRBS. After passing the sample quality test, the DNA samples were treated with a methylation-insensitive restriction enzyme. The digested DNA fragments then underwent end repair; were tagged with an A-tail; and ligated to sequencing adapters, where all cytosines were methylated. The gel was then sliced, and DNA fragments ranging from 150 to 300 bp in length were selected. Subsequently, bisulfite conversion was carried out using the EZ DNA Methylation-Gold Kit (Zymo Research Corp., Irvine, CA, USA). Following bisulfite treatment, unmethylated cytosines are converted to uracil (which becomes thymine after polymerase chain reaction (PCR) amplification), while methylated cytosines remain unchanged. Subsequently, PCR amplification was performed to generate the final DNA library. After library construction, preliminary quantification was conducted using a Qubit 2.0 fluorometer (Thermo Fisher Scientific, Cleveland, OH, USA), and the library was diluted to 1 ng/µL. The Agilent 2100 bioanalyzer was then used to determine the length of the inserted fragments in the library. After meeting the expected fragment size, quantitative PCR (qPCR) was used to accurately quantify the effective concentration of the library (effective concentration > 2 nM) to ensure its quality. After passing inspection, the libraries were pooled according to the required effective concentration and target data volume for Illumina HiSeq sequencing. The fundamental principle of sequencing used is sequencing via synthesis. Four fluorophore-linked 2′-deoxyribonucleoside-5′-triphosphates (dNTPs), DNA polymerase, and adapter primers are introduced into the sequencing flow cell for amplification. During the extension of complementary strands in each sequencing cluster, the incorporation of each fluorescent-labeled dNTP emits its corresponding fluorescence. The sequencing instrument captures these fluorescence signals and, with the aid of computer software, converts them into sequencing peaks, thereby acquiring the sequence information of the target fragment. FastQC was employed to conduct preliminary quality assessments on the raw reads following data extraction. Trimmomatic software (version 0.36) was used to remove sequencing adapters and low-quality data from the sequencing reads, resulting in clean data for subsequent analyses. Bsmap software (version 2.90) was used to align the methylation data with the hg19 reference genome for comparative analysis. This software utilizes a binary segmentation algorithm combined with double statistical tests (Mann–Whitney U test and 2D Kolmogorov–Smirnov test) to quickly achieve DMR detection between paired samples or between two groups of samples. Finally, multiple testing corrections were applied to account for differences in methylation regions. CG sites were used to search for differentially methylated regions. The array data for RRBS are available at GEO under accession number GSE269977.

### 2.4. Methylation EPIC 850k BeadChip Array

Further analysis was conducted using the 850k Methylation EPIC BeadChip (Illumina, San Diego, CA, USA) on 6 pairs of blood samples to validate the DNA methylation landscape observed in 10 pairs of samples from the RRBS. DNA was treated with a bisulfite methylation conversion kit (Zymo Research Corp., Irvine, CA, USA). Then, 0.1 N NaOH was added to the sample to denature the DNA into a single strand, followed by the addition of whole-genome amplification reagents after neutralization. The incubation time was adjusted to optimize the fragmentation effect based on the integrity of the original RNA. The samples were incubated overnight at a constant temperature of 37 °C, resulting in the total amount of amplified DNA reaching 2000–3000 times the initial sample size. The amplified product underwent controlled enzymatic hydrolysis to produce fragmented DNA, eliminating the need for gel electrophoresis. DNA fragments were precipitated with isopropanol and then enriched by centrifugation at 4 °C to achieve purification. After air-drying, the precipitated DNA was dissolved by adding hybridization buffer reagent. The resuspended DNA sample was hybridized with the prepared chip and placed in a hybridization furnace for an overnight reaction. During the hybridization process, the fragmented DNA undergoes denaturation and annealing with site-specific bases, which then attach to the microbeads on the chip. Unhybridized and non-specifically hybridized DNA were both washed off for subsequent staining and extension. Using the captured DNA as a template, a single-base extension reaction was conducted on the chip. Detectable tag groups were then applied, enabling the differentiation of methylation levels at CpG sites within the sample. The processed chip was then placed into a scanner to capture the fluorescence emitted by these groups, resulting in high-resolution images. The length of the regions is limited to a maximum of 300 bp. The array data from the Illumina Methylation EPIC 850k BeadChip can be accessed on Gene Expression Omnibus (GEO) with the accession number GSE268584.

### 2.5. Validation of Methylation by Targeted Bisulfite Sequencing (TBS)

Targeted bisulfite sequencing (TBS), which combines classical bisulfite sequencing PCR (BSP) with high-throughput sequencing, was used in this study to validate 44 large sample pairs of methylated fragments in the target region. First, BS-PCR primers specific to the target region or site were designed and synthesized. Simultaneously, the sample DNA was extracted, and once it passed the required quality control, it was subjected to bisulfite treatment using the EZ DNA Methylation-Gold Kit (Zymo Research Corp., Irvine, CA, USA). Following the treatment, unmethylated cytosine bases were converted to uracil (and to thymine after PCR amplification), while methylated cytosine bases retained their original state. BSP amplification was conducted on the bisulfite-treated template using a high-fidelity DNA polymerase resistant to uracil bases. Amplification products from the same sample’s BSP were then pooled and amplified with labeled primers, followed by the attachment of Illumina sequencing adapters. Basic statistics on the yield and quality of data post-sequencing were first determined, using Trimmomatic-0.36 software to remove sequencing adapters, low-quality bases, and reads, thereby yielding clean reads. Subsequently, the clean reads were aligned with the target sequences using the methylation sequencing data alignment software BSMAP (version 2.73). The methylation level of CG bases on each target sequence of each sample was calculated using the following formula: Methylation level at site C = Number of reads supporting methylation/(Number of reads supporting methylation + Number of reads supporting non-methylation). Additionally, the median methylation level for each site across all samples was determined. A sequencing depth of less than 20X was considered a null value. A t-test was used to detect differences at each locus of each amplicon in each comparison group. The average methylation level of each amplicon was calculated, and the methylation distribution between groups was displayed using box plots and bee swarm plots. An inter-group difference analysis was then performed. The primer sequences are listed in Table S1.

### 2.6. Immunohistochemical Analyses

Immunohistochemical staining was utilized to evaluate AZU1 expression. Rats were housed under two distinct conditions—normobaric normoxia and hypobaric hypoxia—for 72 h, after which they were euthanized under anesthesia. Rat lung tissues were fixed in 10% neutral formaldehyde and processed according to the standard operating procedure (SOP) for pathological examination. The tissues then underwent dehydration, trimming, embedding, sectioning, staining, mounting, and microscopic examination. Images of the sections were captured using the Pannoramic 250 digital slide scanner (3DHISTECH, Budapest, Hungary), facilitating the observation of specific pathological changes. The immunohistochemistry negative control protocol employed in this study utilized adjacent tissue sections from the same specimen, with primary antibody substituted by PBS buffer. All subsequent processing and staining procedures remained identical to those applied to test sections.

### 2.7. Cell Culture

The human umbilical vein endothelial cells (HUVEC) used in this study were purchased from Shanghai Zhongqiao Xinzhou Biotechnology Co., Ltd., and identified by the GMP Laboratory of Shanghai Oriental Hospital (Dongfang Hospital Affiliated to Tongji University) (Shanghai, China). This study cultivated HUVEC under normoxic conditions (5% CO_2_, 21% O_2_, 101 kPa) and hypoxic conditions (5% CO_2_, 1% O_2_, 40 kPa) for 24 h. Hypoxic exposure was simulated in a chamber provided by Hangzhou Aibo Instrument Co., Ltd., Hangzhou, China. HUVEC were cultured in Dulbecco’s Modified Eagle Medium (DMEM) supplemented with 10% fetal bovine serum (FCS) and 1% glutamine (Gibco, Carlsbad, CA, USA).

### 2.8. Quantitative Real-Time Polymerase Chain Reaction (qRT-PCR)

Total RNA was extracted using TRIzol (Invitrogen, Carlsbad, CA, USA). RNA concentration was measured using a NanoDrop 2000 spectrophotometer (NanoDrop Technologies, Wilmington, DE, USA). RNA was reverse-transcribed into complementary DNA (cDNA) using the StarScript II First Strand cDNA Synthesis Kit II (GenStar, Beijing, China). qRT-PCR was performed using a 2× RealStar Green Fast Mix and ROX (GenStar, Beijing, China) on a 384-well qRT-qPCR device (ABI 7500; Applied Biosystems, Waltham, MA, USA). The 2^−ΔΔCT^ method with GAPDH as an internal reference was used to determine gene expression levels. Results are expressed as mean ± SE and were plotted using GraphPad Prism software (version 10.0). The primer sequences are listed in Table S2.

### 2.9. Protein Extraction and Western Blot (WB) Analysis

Cells were collected and lysed on ice with protease inhibitors in Radio Immunoprecipitation Assay (RIPA) buffer (Beyotime, Shanghai, China) for 30 min, followed by centrifugation at 14,000× *g* for 10 min at 4 °C. Protein concentration was quantified using the Bicinchoninic Acid (BCA) Protein Assay Kit (Thermo Fisher Scientific Inc., Waltham, MA, USA). The protein was diluted in 4× loading buffer (TransGen, Beijing, China) and boiled at 100 °C for 5 min. An equal amount of protein was loaded onto a 10% SDS-PAGE gel for electrophoresis and then transferred to a polyvinylidene fluoride (PVDF) membrane (Millipore, Billerica, MA, USA). The membrane was blocked with 5% skim milk powder at room temperature for 1 h and then incubated overnight at 4 °C with the following primary antibodies. After washing three times, the membrane was incubated with the secondary antibody (1:5000 dilution; Abcam, Cambridge, MA, USA) for 2 h. The membrane was then visualized using an enhanced chemiluminescence (ECL) substrate (Thermo Fisher Scientific Inc. Waltham, MA, USA) for development. Exposure and photography were processed using the Tanon fully automatic image analyzer.

### 2.10. Lentivirus Transfection

AZU1 overexpressing and knockdown lentiviruses were purchased from GeneChem (Shanghai, China). HUVEC were transfected with the lentiviruses in the presence of 10 μg/mL polybrene. Puromycin (Sigma-Aldrich, Louis, MO, USA) was used for one week to select stably transfected cells. qRT-PCR and WB analyses confirmed successful transfection.

### 2.11. Tube Formation Assay

The tube formation assay was utilized to assess the ability of endothelial cells to form blood vessels. Matrigel (BD Biosciences, Franklin Lakes, NJ, USA) and DMEM were blended in a 1:1 ratio. A combined volume of 50 µL of this mixture was introduced into each well of a 96-well plate and incubated in a cell culture incubator for 30 min. HUVEC were seeded onto the Matrigel-coated wells and incubated at 37 °C for 3 h. Subsequently, the formation of tubules was examined with an inverted phase contrast microscope, and the extent of tubule formation was assessed by counting the number of meshes and measuring tube length.

### 2.12. Lactate Dehydrogenase Assay (LDH)

After a 24 h exposure period, the conditioned medium was collected and centrifuged at 14,000× *g* for 20 min to remove any cell debris. LDH levels were analyzed using the Cytotoxicity Assay Kit PLUS (LDH; Roche, Basel, Switzerland).

### 2.13. Cell Proliferation Assay

To assess cell proliferation, the Cell Light 5-ethynyl-2′-deoxyuridine (EdU) DNA imaging kit (Riobio, Guangzhou, China) was used for EdU incorporation experiments. All procedures were strictly followed according to the instructions provided with the kit. The ratio of EdU-stained cells (red fluorescence) to Hoechst-stained cells (blue fluorescence) was used to evaluate cell proliferation activity. For the Cell counting Kit-8 (CCK8) cytotoxicity assay, cells were seeded in a 96-well plate, processed according to the manufacturer’s instructions (CCK8 Dojindo CK04-05), and quantified at 450 nm using a ThermoMax microplate reader (Molecular Devices, Sunnyvale, CA, USA).

### 2.14. Assessment of Oxidative Stress Markers in HUVEC

#### 2.14.1. Reactive Oxygen Species (ROS) Fluorescence Detection

According to the manufacturer’s instructions, the CellROX Green Reagent (Thermo Fisher Scientific, C10444) was used to detect ROS. HUVEC were seeded into a 24-well plate and treated under hypoxic and normoxic conditions for 24 h to induce ROS production. Following oxidative stress induction, the accumulation of ROS in stained live cells was measured using a fluorescence microscope with 5 μM CellROX Green Reagent. Quantitative analysis of ROS was performed using ImageJ software (version 1.54).

#### 2.14.2. Glutathione (GSH), Glutathione Oxydized (GSSG), Superoxide Dismutase (SOD), and Malondialdehyde (MDA)

According to the manufacturer’s protocol, the total glutathione, GSH, and GSSG concentrations in cells were measured using the GSH/GSSG ratio detection assay kit (Abcam, Cambridge, MA, USA). The concentration of MDA was detected with an MDA assay kit (Beijing Solarbio Life Sciences, Bejing, China). SOD levels were determined using the SOD Detection Kit (Beyotime Biotechnology, Nantong, China). Each experiment was performed in triplicate to ensure statistical robustness and reproducibility of the results.

### 2.15. Bioinformatics Analysis

Methylation profiling was performed using R v4.2.1 with the ChAMP package to process raw IDAT files. Following initial probe filtering, between-sample signal normalization was executed via the BMIQ algorithm to generate comparable β-values (representing methylation levels) for downstream analysis. Singular-Value Decomposition (SVD) of normalized β-values systematically evaluated technical covariates, detecting potential batch effects that would necessitate correction using ComBat from the SVA package. Concurrent quality assessment included principal component analysis (PCA) and hierarchical clustering.

## 3. Results

### 3.1. Comparison of Whole-Genome DNA Methylation Between HAPE Patients and Control Subjects

When the bisulfite conversion efficiency of RRBS sequencing in 20 samples exceeds 99%, it suggests the reliability of the calculated methylation level at the cytosine (C) site (Figure S1a). The coverage depth exceeds 5×, and the proportions of methylated C sites across different sequence contexts (CG, CHH, and CHG, where H represents A, C, or T) are illustrated in Figure S1b. In the CG context, the proportion of methylated C sites consistently exceeds 60%. Pearson correlation analysis revealed a strong correlation among the samples, indicating high consistency in methylation levels. As the correlation coefficient approaches 1, it indicates increasing similarity in methylation patterns among the samples (see Figure S1c). Methylation levels within the functional regions of two sets of genes were analyzed. The results indicated no significant difference in the average methylation levels of the C site across different genomic functional regions between the HAPE group and the control group for each context (Figure S1d). The gene functional regions encompass the upstream 2 kb, exons, introns, downstream 2 kb, CpG islands (CGI), CGI shores, repeats, and others. Notably, the promoter region extends 2 kb upstream from the transcription start site (TSS). The length distribution of DMRs indicates a typical length of 200 bp (see Figure S1e). This finding suggests the reliability of the RRBS experimental results and the appropriateness of the sample selection. Basic information about the population is listed in Table S3.

**Figure 2:**
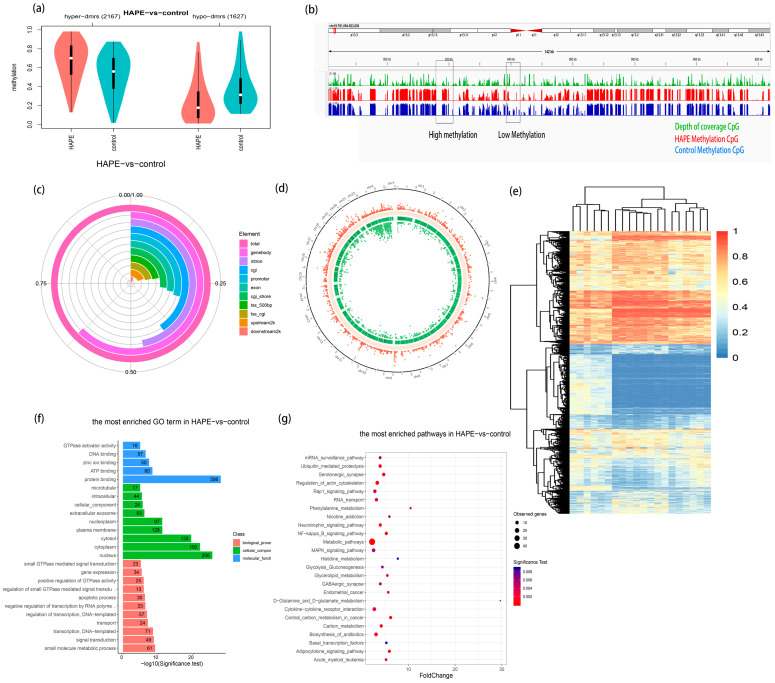
The whole-genome DNA methylation profile of High-altitude pulmonary edema (HAPE) by Reduced-Representation Bisulfite Sequencing (RRBS). (**a**) Violin plot of the average methylation level distribution in differentially methylated regions (DMRs). Hyper-dmr refers to DMRs with high methylation in the sample, while hypo-dmr represents DMRs with low methylation in the sample. (**b**) Import rendering of IGV visualization file in DMR area. (**c**) Distribution proportion of DMR in anchoring area. (**d**) Circos diagram of the distribution of DMR in RRBS sequencing between HAPE group and control group. From the outside to the inside, the scatter plot of hyper-DMR distribution is displayed, facing outward, where points closer to the outer edge indicate higher significance; the GC content heatmap of the sequence, where darker colors represent higher GC content; the gene density heatmap of the sequence, where darker colors represent higher gene content; and the scatter plot of hypo-DMR distribution shows an inward direction, indicating that the more points point inward, the higher the significance. (**e**) A heatmap of DMRs by RRBS between HAPE group and control group. Red indicates hypermethylated, and blue indicates hypermethylated. (**f**) Gene Ontology (GO) terms’ functional enrichment. (**g**) Kyoto Encyclopedia of Genes and Genomes (KEGG) pathways’ functional enrichment.

This study utilized RRBS to perform whole-genome DNA methylation sequencing on the peripheral blood of 10 HAPE patients and 10 control subjects. A total of 3794 DMRs with statistical significance were identified, including 2167 hypermethylated DMRs and 1627 hypomethylated DMRs (Figure 2a). The DMR information was saved in BED format and imported into the Integrative Genomics Viewer (IGV), alongside the methylation information file, for comparative review, as illustrated in Figure 2b. A statistical plot of the distribution of DMR quantities in anchoring areas (such as promoter, exon, intron, CGI, CGI shore, repeat, transcription start site (TSS), transcription end site (TES), etc.) was generated, as shown in Figure 2c. Within this context, the proportions are as follows: promoter, 29.20%; upstream 2 kb, 19.56%; exon, 28.10%; intron, 46.97%; downstream 2 kb, 6.93%; CGI, 40.41%; and CGI shore, 27.02%. Figure 2d illustrates the distribution and significance analysis of DMRs on the stained chromosomes (corrected *p*-value < 0.05). Figure 2e presents the heatmap results of the significantly different DMRs in the RRBS sequencing. Subsequently, Gene Ontology (GO) and the Kyoto Encyclopedia of Genes and Genomes (KEGG) functional enrichment analyses were conducted on the genes related to DMRs. GO enrichment analysis results showed that the most enriched GO terms were “regulation of transcription”, “small molecule metabolic process”, “signal transduction”, “positive regulation of transcription by RNA polymerase II”, and “gene expression” (Figure 2f). KEGG enrichment analysis results showed that “metabolic pathways”, “PI3K-Akt signaling pathway”, and “MAPK signaling pathway” were significantly enriched (Figure 2g).

**Figure 3:**
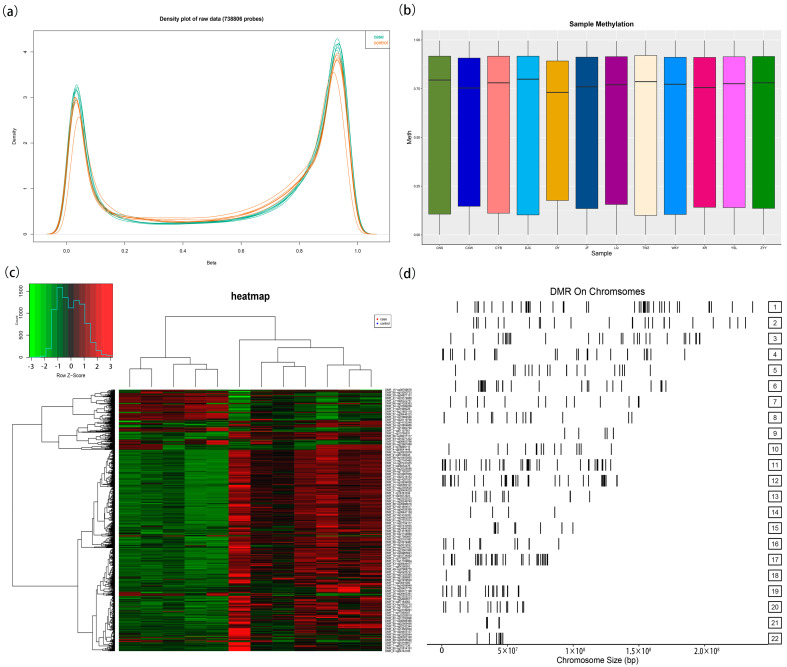
The whole-genome DNA methylation profile of HAPE by 850k EPIC BeadChip. (**a**) Normalized density distribution map of methylation degree of each sample probe. The horizontal axis represents the degree of methylation, and the vertical axis represents the probe density corresponding to the degree of methylation. Each curve represents a sample. Green represents the HAPE case group, and red represents the control group. (**b**) Boxplot of methylation levels for each sample. (**c**) A heatmap of DMRs by 850k EPIC BeadChip between HAPE group and control group. Red indicates hypermethylated, and green indicates hypermethylated. (**d**) Chromosome distribution map of DMRs by 850k EPIC BeadChip between HAPE group and control group.

Furthermore, this study utilized the Illumina EPIC 850k BeadChip to sequence DNA methylation in the peripheral blood of six HAPE patients and six control subjects. Figure 3a displays the density distribution of methylation levels for each sample’s probes after normalization, revealing a balanced distribution between the two groups. Figure 3b demonstrates minimal differences in the methylation levels among the samples, indicating comparable signal strengths. The 850k sequencing data exhibited a relatively high quality. Differential DMR analysis revealed that 433 DMRs were statistically significant (|Δβ| > 0.1, *p* < 0.05). Figure 3c presents the DMR heatmap derived from the 850k BeadChip sequencing, while Figure 3d illustrates the chromosomal distribution of the DMRs.

### 3.2. Targeted Bisulfite Sequencing Identifies Key Methylation Driver Genes

**Figure 4:**
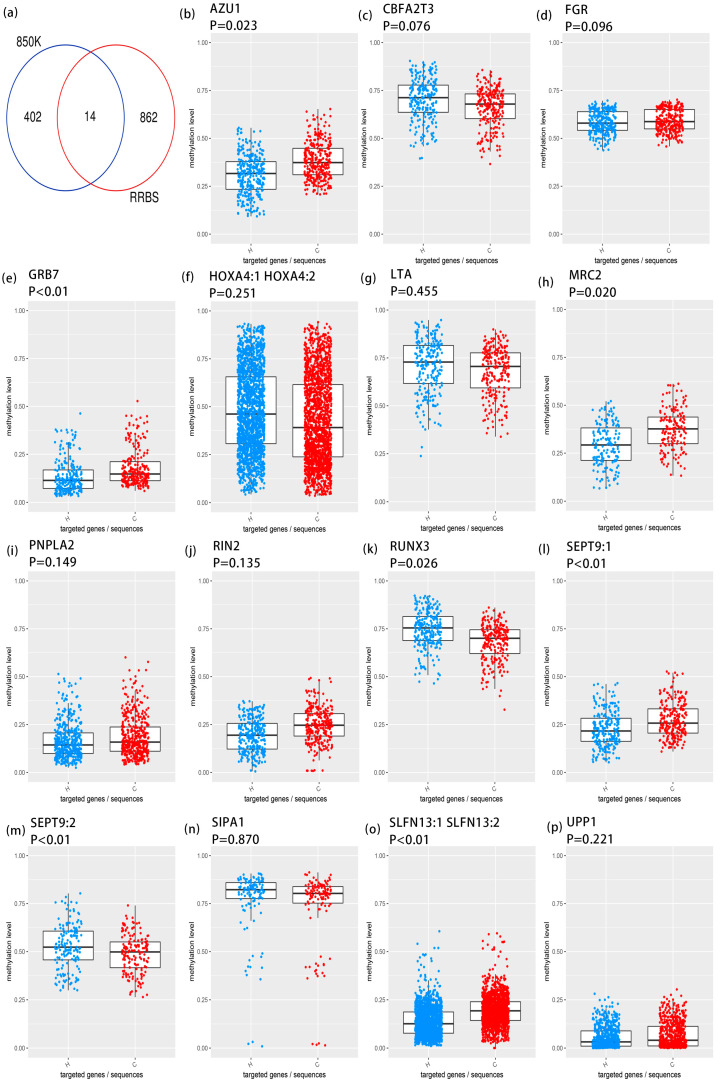
Targeted bisulfite sequencing identifies key methylation driver genes. (**a**) The Venn plot of intersection between RRBS sequencing and 850k EPIC BeadChip. (**b**–**p**) Box plot and colony plot display the inter-group differences of 14 genes.

By intersecting the results of 850k sequencing and RRBS sequencing, we identified 18 DMRs located on the following 14 genes: FGR proto-oncogene, Src family tyrosine kinase (FGR), RUNX3, GRB7, Ras and Rab interactor 2 (RIN2), signal-induced proliferation-associated 1 (SIPA1), SEPT9, MRC2, AZU1, homeobox A4 (HOXA4, schlafen family member 13 (SLFN13), uridine phosphorylase 1 (UPP1), lymphotoxin alpha (LTA), patatin-like phospholipase domain containing 2 (PNPLA2), and CBFA2/RUNX1 partner transcriptional co-repressor 3 (CBFA2T3) (Figure 4a). Figure S2 presents the methylation value plot for each probe site within the differential methylation regions of 14 genes in the 850k sequencing results. Moreover, TBS sequencing validation was conducted on the differential DMRs in the promoter regions of 14 genes across 88 samples (44 pairs). Among these, the DMR methylation levels of five genes demonstrated statistical significance in the TBS validation results (*p* < 0.05): AZU1, GRB7, MRC2, RUNX3, and SEPT9 (Figure 4b–p). AZU1 has piqued our keen interest because previous research indicates that it is a crucial gene influencing the severity of acute mountain sickness (AMS) [10]. Consequently, we undertook experimental exploration of the mechanism of action of AZU1 in subsequent studies.

### 3.3. Verification of AZU1 Expression and Construction of an Acute Hypobaric Hypoxic Cell Injury Model

**Figure 5:**
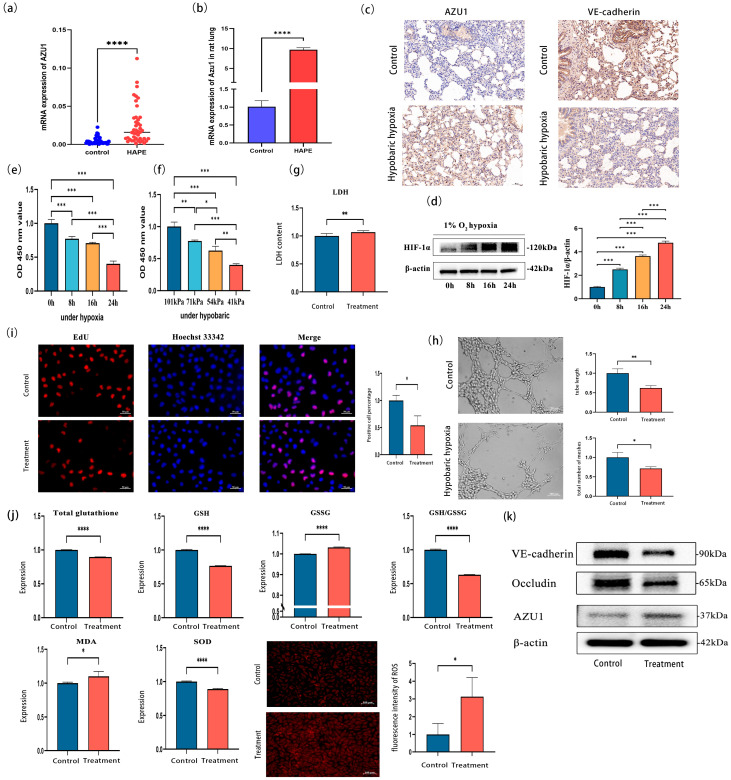
Verification of azurocidin 1 (AZU1) expression and construction of acute hypobaric hypoxic HUVEC model. (**a**) Scatter plot of the AZU1 expression in blood samples between the HAPE group and the control group. (**b**) The AZU1 expression in rat lung between the HAPE group and the control group. (**c**) Immunohistochemical staining of rat lung AZU1 and VE-cadherin expression (immunohistochemistry, ×20). (**d**) Expression of HIF-1 α in Human Umbilical Vein Endothelial Cells (HUVEC) after 1% O_2_ treatment at different cultivation times (0 h, 8 h, 16 h, and 24 h). (**e**) Cell viability of HUVEC at different time points for 24 h. (**f**) Cell viability of HUVEC cultured under different pressures for 24 h (with 1% O_2_). (**g**) Acute hypobaric hypoxia leads to increased Lactate Dehydrogenase Assay (LDH) release in HUVEC. (**h**) Acute hypobaric hypoxia inhibits HUVEC angiogenesis (tube length and branching points). (**i**) EdU assay of HUVEC between treatment group and control group. Scale bar, 50 μm. (**j**) Differences in oxidative stress indicators between two groups. Oxidative stress indicators: total glutathione, Glutathione (GSH), Glutathione Oxydized (GSSG), Superoxide Dismutase (SOD), and Malondialdehyde (MDA) and Reactive Oxygen Species (ROS). ROS levels were detected by fluorescence intensity. Scale bar, 100 μm. (**k**) Western blot (WB) of HUVEC of AZU1, VE-cadherin, and occludin between treatment group and control group. * *p* < 0.05, ** *p* < 0.01, *** *p* < 0.001, and **** *p* < 0.0001.

To further verify the differential expression of AZU1 mRNA, qRT-PCR was conducted on 88 blood samples. The results revealed a statistically significant difference in AZU1 expression levels between the HAPE group and the control group (Figure 5a). qRT-PCR and immunohistochemical analysis revealed that AZU1 expression in the lung tissue of rats subjected to hypobaric hypoxia was significantly higher than in rats maintained under normobaric normoxia (Figure 5b,c). Moreover, immunohistochemical analysis revealed reduced expression of endothelial junction proteins (VE-cadherin), suggesting impaired barrier integrity in rat endothelial cells following hypobaric hypoxia exposure (Figure 5c). Negative control images are provided in Figure S3.

The phenotype and function of endothelial cells are closely related to the occurrence and development of HAPE. When the human body is exposed to a hypoxic environment for a short period of time, decompensation may occur, leading to vascular damage and initial edema [11]. Inflammatory factors, once released, act on endothelial cells, increasing their permeability and exacerbating the degree of edema. Therefore, we established a hypobaric hypoxia injury model using HUVEC to investigate the effects of AZU1 on vascular endothelial cells and to explore its mechanism of action in the occurrence and development of HAPE.

According to existing research, a 1% O_2_ environment has been widely used to construct cellular hypoxia models [12]. To explore the optimal duration of hypoxia cultivation, four time points were set up in this study: 0 h, 8 h, 16 h, and 24 h. The results showed that the expression of HIF-1α, a marker of hypoxia, increased with the duration of hypoxia culture (Figure 5d). Additionally, low-oxygen cultivation for 8 h, 16 h, and 24 h resulted in lower cell viability compared to the normoxic group (Figure 5e). This study established four atmospheric pressure conditions: 101 kPa, 71 kPa, 54 kPa, and 41 kPa. As shown in the results, the cell viability of HUVEC was impaired under all three lower pressures (Figure 5f). Therefore, we ultimately chose 1% O_2_, 41 kPa, and 24 h of cultivation as the conditions for the acute hypobaric hypoxia model in this study, referred to as the treatment group. The control group was cultured under conditions of 21% O_2_, 101 kPa, and 24 h. The research results showed that, compared with the control group, the LDH release in HUVEC in the treatment group increased (t = 3.80, *p* < 0.01) (Figure 5g). The tube length (t = −5.10, *p* = 0.01) and branching points (t = −3.64, *p* = 0.02) of HUVEC blood vessels under acute hypobaric hypoxia were lower than those in the control group, indicating that acute hypobaric hypoxia inhibited HUVEC angiogenesis (Figure 5h). The EdU experiment results showed that acute hypobaric hypoxia inhibited the proliferation of HUVEC (t = −3.99, *p* = 0.02) (Figure 5i). In addition, Figure 5j shows that acute hypobaric hypoxia caused oxidative stress damage to HUVEC. In the treatment group, total glutathione in HUVEC decreased (t = −45.41, *p* < 0.001), GSSG content increased (t = 25.47, *p* < 0.001), GSH levels decreased (t = −44.05, *p* < 0.001), and the GSH/GSSG ratio decreased (t = −46.33, *p* < 0.001). Concurrently, the SOD expression level in the treatment group significantly decreased (t = −17.79, *p* < 0.001), malondialdehyde levels significantly increased (Z = 2.62, *p* < 0.01), and ROS levels significantly increased (t = 2.98, *p* = 0.04). Western blot analysis demonstrated enhanced cellular permeability concomitant with elevated AZU1 expression following hypobaric hypoxia exposure (Figure 5k).

### 3.4. AZU1 Promotes HUVEC Injury Induced by Acute Hypobaric Hypoxic

Building on the established cell damage model, we utilized lentiviral technology to construct stable HUVEC cell lines with downregulated and overexpressed AZU1. We investigated the role of AZU1 in HUVEC damage induced by acute hypobaric hypoxic exposure. WB results showed that the sh_125572 virus significantly inhibited AZU1 expression in HUVEC under acute hypobaric hypoxia, demonstrating that the construction of HUVEC cell lines with knocked-down AZU1 expression was successful and stable (Figure S4a,b). Subsequent experiments were conducted using HUVEC cell lines transfected with the sh_125572 virus to knock down AZU1 expression (sh_AZU1). Figure S4c,d demonstrate the successful and stable construction of HUVEC cell lines overexpressing AZU1.

**Figure 6:**
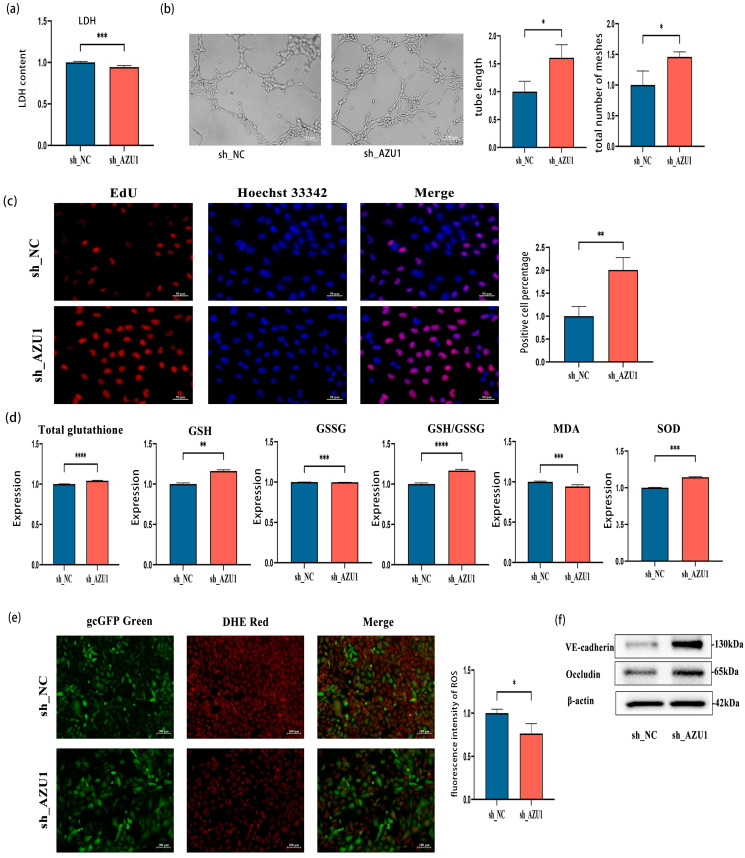
Low expression of AZU1 reduces the damage of acute hypobaric hypoxia to HUVEC. (**a**) The release of LDH in HUVEC with knockdown AZU1 expression under acute hypobaric hypoxia. (**b**) Knocking down AZU1 expression promotes the angiogenic ability of HUVEC (tube length and branching points) under acute hypobaric hypoxia. (**c**) EDU experiment shows that knocking down AZU1 expression promotes the proliferation of HUVEC. Scale bar, 50 μm. (**d**,**e**) Knocking down AZU1 expression reduces oxidative stress damage to HUVEC caused by acute hypobaric hypoxia. Oxidative stress indicators: total glutathione, GSH, GSSG, GSH/GSSG, MDA, SOD, and ROS. ROS levels were detected by fluorescence intensity. Scale bar, 100 μm. (**f**) Western blot of VE-cadherin and occludin between low expression of AZU1 group and control group. * *p* < 0.05, ** *p* < 0.01, *** *p* < 0.001, and **** *p* < 0.0001.

Subsequent research found that knocking down AZU1 resulted in a decrease in LDH levels in HUVEC in the treatment group (t = −6.26, *p* < 0.001) (Figure 6a). The tube length (t = 3.51, *p* = 0.03) and branching points (t = 3.26, *p* = 0.03) of the sh-AZU1 group were significantly increased compared to the sh-NC group under acute hypobaric hypoxia (Figure 6b). The EdU experiment results showed that knocking down AZU1 in an acute hypobaric hypoxic environment promoted the proliferation of HUVEC cell lines (t = 5.08, *p* = 0.01) (Figure 6c). Figure 6d,e indicate that knocking down AZU1 expression alleviated oxidative stress damage to HUVEC caused by acute hypobaric hypoxia. Compared with the sh-NC group, the total glutathione content increased (t = 16.41, *p* < 0.001), GSSG content decreased (t = −10.38, *p* < 0.001), GSH content increased (Z = 2.88, *p* < 0.01), and the GSH/GSSG ratio increased (t = 19.18, *p* < 0.001) under acute hypobaric hypoxia in the sh-AZU1 group. Concurrently, under acute hypobaric hypoxia, the MDA content in the sh-AZU1 group decreased compared to the sh-NC group (Z = −2.95, *p* < 0.01), while the SOD content in the sh-AZU1 group increased compared to the sh-NC group (t = 42.85, *p* < 0.001). Figure 6e shows that the ROS content in the sh-AZU1 group decreased compared to the sh-NC group (t = −3.31, *p* = 0.03). Western blot analysis demonstrated that knockdown of AZU1 alleviated the increased cell permeability induced by exposure to hypobaric hypoxia (Figure 6f).

**Figure 7:**
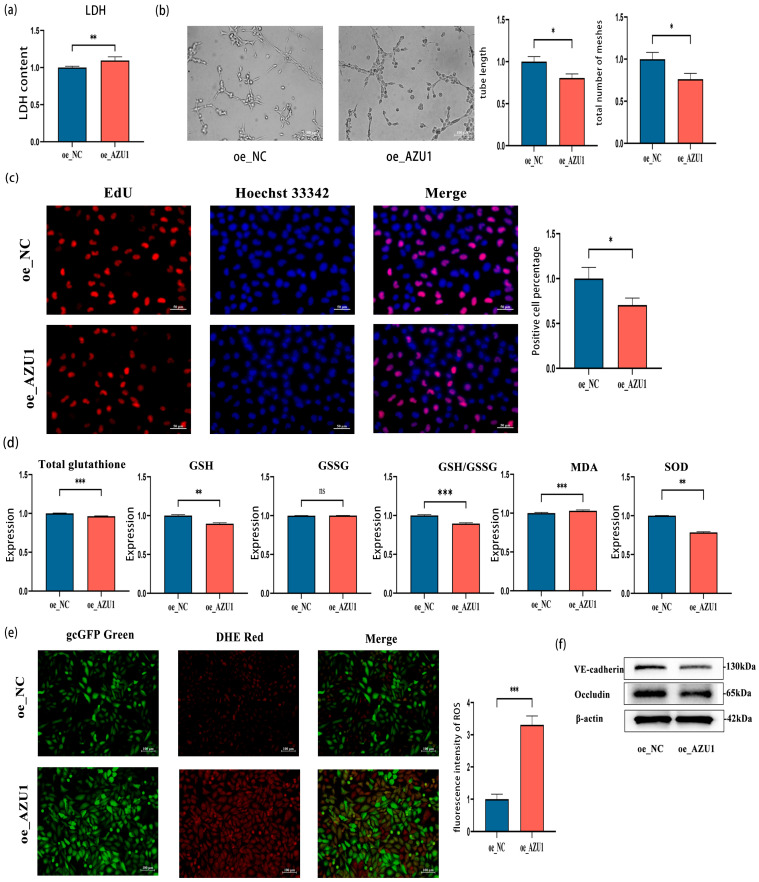
High expression of AZU1 aggravates the damage of acute hypobaric hypoxia to HUVEC. (**a**) The release of LDH in HUVEC with overexpressing AZU1 under acute hypobaric hypoxia. (**b**) Overexpressing AZU1 reduces the angiogenic ability of HUVEC (tube length and branching points) under acute hypobaric hypoxia. (**c**) EDU experiment shows that overexpressing AZU1 reduces the proliferation of HUVEC. Scale bar, 50 μm. (**d**,**e**) Overexpressing AZU1 promotes oxidative stress damage to HUVEC caused by acute hypobaric hypoxia. Oxidative stress indicators: total glutathione, GSH, GSSG, GSH/GSSG, MDA, SOD, and ROS. ROS levels were detected by fluorescence intensity. Scale bar, 100 μm. (**f**) Western blot of VE-cadherin and occludin between high expression of AZU1 group and control group. * *p* < 0.05, ** *p* < 0.01, and *** *p* < 0.001, ns means not significant.

After hypobaric hypoxia treatment, the cell phenotype of AZU1-overexpressing cell lines showed opposite results compared to AZU1 knockdown cell lines. Figure 7a–f demonstrate that AZU1 overexpression in hypoxic HUVEC significantly elevates LDH release and cellular permeability while suppressing proliferation and augmenting oxidative stress. Therefore, this study found that knocking down the expression of AZU1 can significantly reduce the damage caused by acute hypobaric hypoxia to HUVEC, while overexpression of AZU1 can exacerbate the damage.

### 3.5. AZU1 Exacerbates HUVEC Injury Model Damage by Activating the P38 MAPK Signaling Pathway

In the KEGG enrichment analysis results shown in Figure 2g, the MAPK signaling pathway was significant. In previous studies, we found that the MAPK signaling pathway is one of the most significantly enriched pathway in differential gene functions [10]. Furthermore, there is limited research on the relationship between AZU1 and the MAPK signaling pathway. Therefore, this study used rescue experiments to explore the mechanism by which overexpression of AZU1 exacerbates HUVEC injury under acute hypobaric hypoxia, using the P38 MAPK inhibitor SB203580.

**Figure 8:**
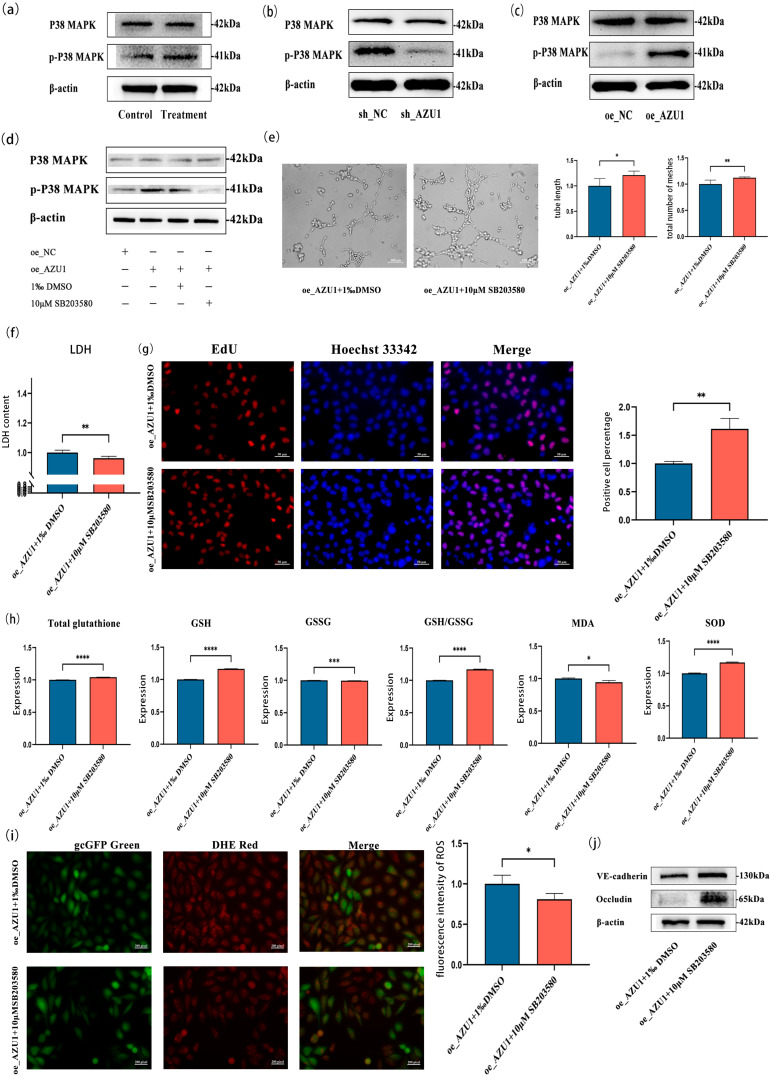
AZU1 exacerbates HUVEC injury model damage by activating the P38 MAPK signaling pathway. (**a**) The expression of P38 MAPK protein between treatment group and control group. (**b**) Knocking down AZU1 increases the expression of P38 MAPK protein in HUVEC under acute hypobaric hypoxia. (**c**) Overexpressing AZU1 decreases the expression of P38 MAPK protein in HUVEC under acute hypobaric hypoxia. (**d**) A 10 μM concentration of P38 MAPK inhibitor reduces protein levels of p-P38 MAPK. (**e**) Inhibition of p-P38 MAPK enhances the angiogenic ability of HUVEC overexpressing AZU1 in acute hypobaric hypoxia. (**f**) Inhibition of p-P38 MAPK reduces the release of LDH in HUVEC with overexpressing AZU1 under acute hypobaric hypoxia. (**g**) EDU experiment shows that inhibition of p-P38 MAPK enhances the proliferation of HUVEC with overexpressing AZU1 under acute hypobaric hypoxia. Scale bar, 50 μm. (**h**–**i**) Inhibition of p-P38 MAPK reduces oxidative stress damage to HUVEC with overexpressing AZU1 caused by acute hypobaric hypoxia. Oxidative stress indicators: total glutathione, GSH, GSSG, GSH/GSSG, MDA, SOD, and ROS. ROS levels were detected by fluorescence intensity. Scale bar, 100 μm. (**j**) Western blot of VE-cadherin and occludin between overexpressing AZU1 group and P38 MAPK inhibitor group. * *p* < 0.05, ** *p* < 0.01, *** *p* < 0.001, and **** *p* < 0.0001.

Acute hypobaric hypoxia significantly induced p-p38 MAPK activation in HUVEC (Figure 8a), whereas AZU1 silencing attenuated this phosphorylation without altering total p38 MAPK levels (Figure 8b). Conversely, AZU1 overexpression markedly enhanced p-p38 MAPK activation independent of total p38 MAPK expression (Figure 8c). Pharmacological inhibition with 10 μM SB203580 effectively neutralized this phosphorylation (Figure 8d) while rescuing cellular viability versus Dimethyl Sulfoxide (DMSO) controls (*p* < 0.01, q = 0.17; Figure S5). Crucially, SB203580 treatment under hypoxic conditions reversed AZU1-mediated angiogenic impairment, evidenced by increased tube formation (*p* = 0.01) and branching complexity (*p* < 0.01) (Figure 8e), concurrently attenuating LDH release in injury models (Figure 8f). EdU assays further confirmed restored proliferation capacity upon p38 inhibition (Figure 8g). Mechanistically, p-p38 MAPK blockade alleviated oxidative stress through coordinated enhancement of glutathione metabolism (↑total glutathione/↑GSH/↓GSSG/↑GSH:GSSG ratio), elevated SOD activity, and reduced MDA accumulation (Figure 8h), ultimately normalizing AZU1-driven ROS overproduction (Figure 8i) and preserving endothelial barrier integrity (Figure 8j).

## 4. Discussion

In this study, we investigated the genome-wide DNA methylation characteristics of HAPE patients and the epigenetic heterogeneity between HAPE patients and control subjects using RRBS and 850K DNA methylation sequencing technologies. Our study revealed a series of significantly differentially methylated gene promoter regions, and the abnormal methylation status in these regions may be a key driving factor in the occurrence of HAPE. Moreover, DNA methylation changes strongly affect the driver genes and pathways of HAPE in a complex manner. After validation, we identified five key methylation-driven genes related to HAPE: AZU1, GRB7, MRC2, RUNX3, and SEPT9. It is interesting that our previously published study has found that AZU1 has high diagnostic value in AMS ^10^, so we chose AZU1 and further validated its role in the pathogenesis of HAPE through experiments. Using RT-qPCR technology, we found that the expression of AZU1 was significantly increased not only in the blood of HAPE patients but also in the lung tissues of rats under hypobaric hypoxia. These findings are consistent with the methylation sequencing results, supporting the key role of AZU1 in HAPE.

AZU1, also known as heparin-binding protein (HBP) or 37 kDa cationic antimicrobial protein, is a neutrophil-derived granule protein [13,14]. As a multifunctional protein, AZU1 has been shown to be associated with various diseases, including sepsis, acute lung injury, and acute respiratory distress syndrome [13,15]. However, the relationship and mechanism of action between AZU1 and HAPE have not yet been fully elucidated.

This study focuses on HUVEC and utilizes various experimental methods to construct an acute hypobaric hypoxia cell injury model, while investigating the relationship between AZU1 and cell phenotypes. The cell phenotypes include LDH release, cell proliferation ability, angiogenesis ability, and oxidative stress damage. Previous studies have shown that hypoxia and oxidative stress damage often coexist, with oxidative stress having a negative impact on cell health [16]. The results of this study are consistent with previous findings, showing an increase in ROS generation, a decrease in SOD levels, an increase in MDA, and a decrease in the GSH/GSSG ratio in cells under hypoxic conditions [17,18,19], indicating that acute hypobaric hypoxia caused oxidative stress damage to HUVEC. Current studies suggest that oxidative stress in blood vessels precedes parenchymal oxidative stress [20]. Therefore, vascular oxidative stress may be a key focus for early treatment and intervention of HAPE, warranting further research. The relationship between vascular endothelial permeability and edema is particularly close in hypoxic environments.

It should be clarified that AZU1 acts as an inflammatory factor [13]. Previous studies have shown that, under conditions of hypoxia or infection, T cells and monocytes release chemokines like IL-8, which are associated with AZU1 [21]. After IL-8 stimulation, neutrophils release AZU1 pre-existing in secretory vesicles and azurophilic granules. The released AZU1 acts on the glycosaminoglycans on the surface of endothelial cells, activating PKC and Rho kinases while allowing Ca^2+^ to enter. The ROS produced by hypoxia promotes the expression of PLCγ, which, together with Ca^2+^, activates PKC [22]. These changes rearrange the endothelial cytoskeleton, leading to increased endothelial permeability and inducing edema, while also allowing neutrophils to affect other tissues and cells [23]. AZU1 may also exacerbate inflammation and oxidative stress damage by upregulating its own expression, activating the NF-κB signaling pathway, inducing IL-6 expression, and leading to kidney injury in mice [24]. The upregulated expression of TNF-α due to the high expression of AZU1 inhibits eNOS activity, leading to a decrease in NO bioavailability. It also induces cell apoptosis, promotes ROS production, and exacerbates oxidative stress damage [25]. This explains why AZU1 in the HUVEC injury model exacerbates reductions in cell viability and oxidative stress damage in the research results. Our findings demonstrate that AZU1 upregulation during hypobaric hypoxia aligns with physiological stress response mechanisms. Interestingly, AZU1 overexpression under normoxic conditions still induced endothelial damage—manifested by reduced viability, impaired proliferation, elevated oxidative stress, and increased permeability (Figure S6)—indicating its pathogenic role in both physiological and acute hypoxic environments. These observations establish AZU1 as a hypoxia-independent regulator of fundamental endothelial pathways, functioning as a core pathological mediator rather than merely executing hypoxia-triggered signaling cascades.

Additionally, the research results indicate that AZU1 may play an important role in the occurrence and development of HAPE through the MAPK pathway. The activation of the MAPK signaling pathway is associated with hypoxia, oxidative stress, and inflammation, and it is involved in regulating these biological processes [26,27,28]. However, there is currently limited research on the relationship between the MAPK pathway, HAPE, and AZU1. This study conducted a recovery experiment using the p38 MAPK inhibitor SB203580. Cell damage caused by overexpression of AZU1 under acute low pressure and hypoxia can be rescued through SB203580, indicating that AZU1 plays a role in endothelial cell damage caused by acute low pressure and hypoxia through the p38 MAPK signaling pathway.

This study integrates interdisciplinary methods, including epigenetics, high-throughput sequencing technology, bioinformatics analysis, and molecular biology experiments, demonstrating the enormous potential of interdisciplinary approaches in revealing complex disease mechanisms. The importance and innovations of this study are mainly embodied in the following aspects. (1) For the first time, RRBS sequencing technology and 850K DNA methylation chip technology were systematically applied in HAPE research. This enables comprehensive identification of DNA methylation abnormalities related to HAPE, filling an important gap in high-altitude environmental disease research. (2) Significant abnormalities in the methylation status of five genes were identified for the first time, indicating their potential importance in the pathogenesis of HAPE. In particular, identifying and focusing on AZU1 provides a new breakthrough for revealing the molecular mechanism of HAPE. (3) This study not only identified potential key genes through sequencing data analysis but also further validated their functional importance through multi-level experiments.

Although this study has made significant progress in identifying key methylation driver genes associated with HAPE, there are still some limitations, including the following aspects: (1) The sample size of this study is relatively limited, which may affect the generalizability and reliability of the results. Due to the difficulty in obtaining HAPE patients and the small sample size, there may be some bias in statistical analysis. Future research needs to increase the sample size to improve the statistical significance and generalizability of the findings. (2) A limitation arises from our observation of reduced angiogenesis under hypoxic-pressure conditions, where a simultaneous 50% loss of cell viability introduces uncertainty regarding whether impaired tube formation reflects specific dysregulation of angiogenic programming or general cellular attrition. Without viability-normalized quantification or death-resistant endothelial models, we cannot fully decouple these confounding factors, potentially overstating hypoxia-specific angiogenic defects. (3) While pharmacological inhibition of p-p38 MAPK with SB203580 provided mechanistic insights, we acknowledge the inherent limitations of small-molecule inhibitors. Although SB203580 exhibits high specificity for p-p38, potential off-target effects cannot be fully excluded. To mitigate this concern, future studies utilizing conditional p38 knockout models or AZU1 knock-in systems would strengthen causal attribution. (4) Meanwhile, the use of HUVEC as an in vitro model, while advantageous for experimental manipulation and establishing controlled conditions, may not fully recapitulate the pathophysiological responses of pulmonary endothelial cells in HAPE. Although we confirmed AZU1 upregulation in HUVEC following hypobaric hypoxia exposure (Figure 5k), future validation in primary pulmonary endothelial cells or ex vivo lung tissue models would strengthen the translational relevance of these findings.

## 5. Conclusions

Overall, AZU1 is a key methylation driver gene that affects the occurrence and development of HAPE. Upregulated AZU1 exacerbates acute hypobaric hypoxia-induced HUVEC injury through the p38 MAPK signaling pathway. This indicates that high-altitude hypobaric hypoxia stimulates hypomethylation of the AZU1 promoter region, leading to an increase in AZU1 expression and activation of the p38 MAPK signaling pathway, which affects pulmonary vascular injury and induces HAPE. The results of this study provide new insights into the molecular mechanisms of HAPE, particularly revealing the crucial role of DNA methylation in regulating gene expression and disease progression. The identified methylation driver gene AZU1 not only offers a potential biomarker for the diagnosis and risk assessment of HAPE but also serves as a target for developing new treatment strategies. Future research can further explore the specific regulatory mechanisms of other methylation driver genes, as well as their variations in different individuals. Additionally, utilizing larger-scale samples and more advanced technologies, such as single-cell methylation sequencing, can more comprehensively reveal the epigenetic regulatory network of HAPE, providing stronger support for personalized healthcare.

## Data Availability

All sequencing data are deposited in the GEO database (https://www.ncbi.nlm.nih.gov/geo/) under accession nos. GSE269977 (accessed on 30 Jun 2025) and GSE268584 (accessed on 29 May 2025).

## Supplementary Materials

The following supporting information can be downloaded at https://www.mdpi.com/article/10.3390/antiox14070835/s1, Figure S1: Genome wide methylation map of RRBS sequence title; Figure S2: Point plot of methylation values for each probe site on the differentially methylated regions of 14 genes in the 850k EPIC BeadChip results; Figure S3: Negative control images of IHC; Figure S4: Screening of stable HUVEC cell lines using lentiviral transfection technology; Figure S5: Cellular viability of HUVEC overexpressing AZU1 at different concentrations of P38 MAPK inhibitors; Figure S6: High expression of AZU1 aggravates the damage of normobaric normoxia to HUVEC; Table S1: The primer sequences of Primer amplification sites for TBS; Table S2: The data of qRT-PCR test; Table S3: Basic information of sequencing objects.
