# Supplementary material for: AZU1 as a DNA Methylation-Driven Gene: Promoting Oxidative Stress in High-Altitude Pulmonary Edema
Source: Antioxidants (Basel). 2025 Jul 8;14(7):835. doi: 10.3390/antiox14070835 (PMC12291896; doi:10.3390/antiox14070835)
Supplement: Supplementary file 1 [file antioxidants-14-00835-s001.zip › antioxidants-3613600-supplementary.docx]

**The supplementary materials**

**Figure S1: Genome wide methylation map of RRBS sequence title.**

Bisulfite **conversion** efficiency evaluation. (b) Average methylation level of each sample. (c) Methylation level map of genomic functional elements between HAPEgroup and control group. The gene functional region encompasses the upstream 2kb, exon, intron, downstream 2kb, CGI, CGI shore, repeat, among others. The promoter region extends 2kb upstream from the TSS site. Each component is evenly divided into 20 windows, and the methylation levels in each window are counted separately. The average value is taken within the group. (d) 20 Samples correlation analysis. Calculate the methylation levels within each bin using a 20Kbp/bin sequencing environment, and then perform Pearson correlation analysis. (b) Histogram of DMR length distribution

**Figure S2: Point plot of methylation values for each probe site on the differentially methylated regions of 14 genes in the 850k EPIC BeadChip results.**

The title represents the differentially methylated region, the horizontal axis represents the number of probes contained in the region (sorted by probe position on the chromosome), the vertical axis represents the degree of methylation of each group sample on the probe, and the upper right corner represents the p-value of the differential analysis of the region in the group. The scatter plot represents the specific methylation degree values of each sample, the solid line plot represents the mean of each group sample on the probe, and the dashed line plot represents the curve fitted using the Loess method.

**Figure S3: Negative control images of IHC.**

**Figure S4: Screening of stable HUVEC cell lines using lentiviral transfection technology.**

1. Western blotting indicates the expression levels of AZU1 in HUVEC after transfection of three lentiviruses under acute hypobaric hypoxia. (b) Quantitative analysis of AZU1 expression in HUVEC after transfection with three types of lentiviruses. (c) Western blotting indicates overexpression expression levels of AZU1 in HUVEC under acute hypobaric hypoxia. (d) Quantitative analysis of overexpression expression levels of AZU1 in HUVEC.

**Figure S5: Cellular viability of HUVEC overexpressing AZU1 at different concentrations of P38 MAPK inhibitors.**

**Figure S6: High expression of AZU1 aggravates the damage of normobaric normoxia to HUVEC.**

(a)The Release of LDH in HUVEC with overexpressing AZU1 under normobaric normoxia.(b) Overexpressing AZU1 reduces the angiogenic ability of HUVEC (tube length and branching points)under normobaric normoxia. (c) EDU experiment shows that Overexpressing AZU1 reduces the proliferationof HUVEC. Scale bar, 50 y m, (d-e) Overexpressing AZU1 promotes oxidative stress damage to HUVEC. oxidativestress indicators: total glutathione, GSH, GSSG, GSH/GSSG, MDA, SOD, ROS. ROS levels were detected byfluorescence intensity, Scale bar, 100 u m. (f Western blot of VE-cadherin and Occludin between high expressionof AZU1 group and control group.* p <0.05,** P <0.01,*** p <0.001.* P <0.05,**p <0.01, *** p <0.001.

Table S1: The primer sequences of Primer amplification sites for TBS.

Table S2: The data of qRT-PCR test.

Table S3: Basic information of sequencing objects.
