# Supplementary figures and images for: AZU1 as a DNA Methylation-Driven Gene: Promoting Oxidative Stress in High-Altitude Pulmonary Edema
Source: Antioxidants (Basel). 2025 Jul 8;14(7):835. doi: 10.3390/antiox14070835 (PMC12291896; doi:10.3390/antiox14070835)

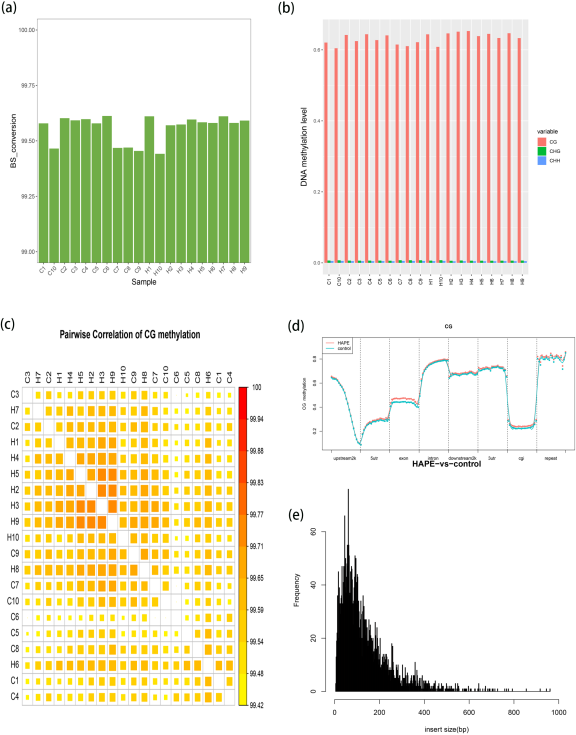

Supplement: Supplementary file 1 [file antioxidants-14-00835-s001.zip › Supplementary figures 1-6/supplemental figure 1.tif]

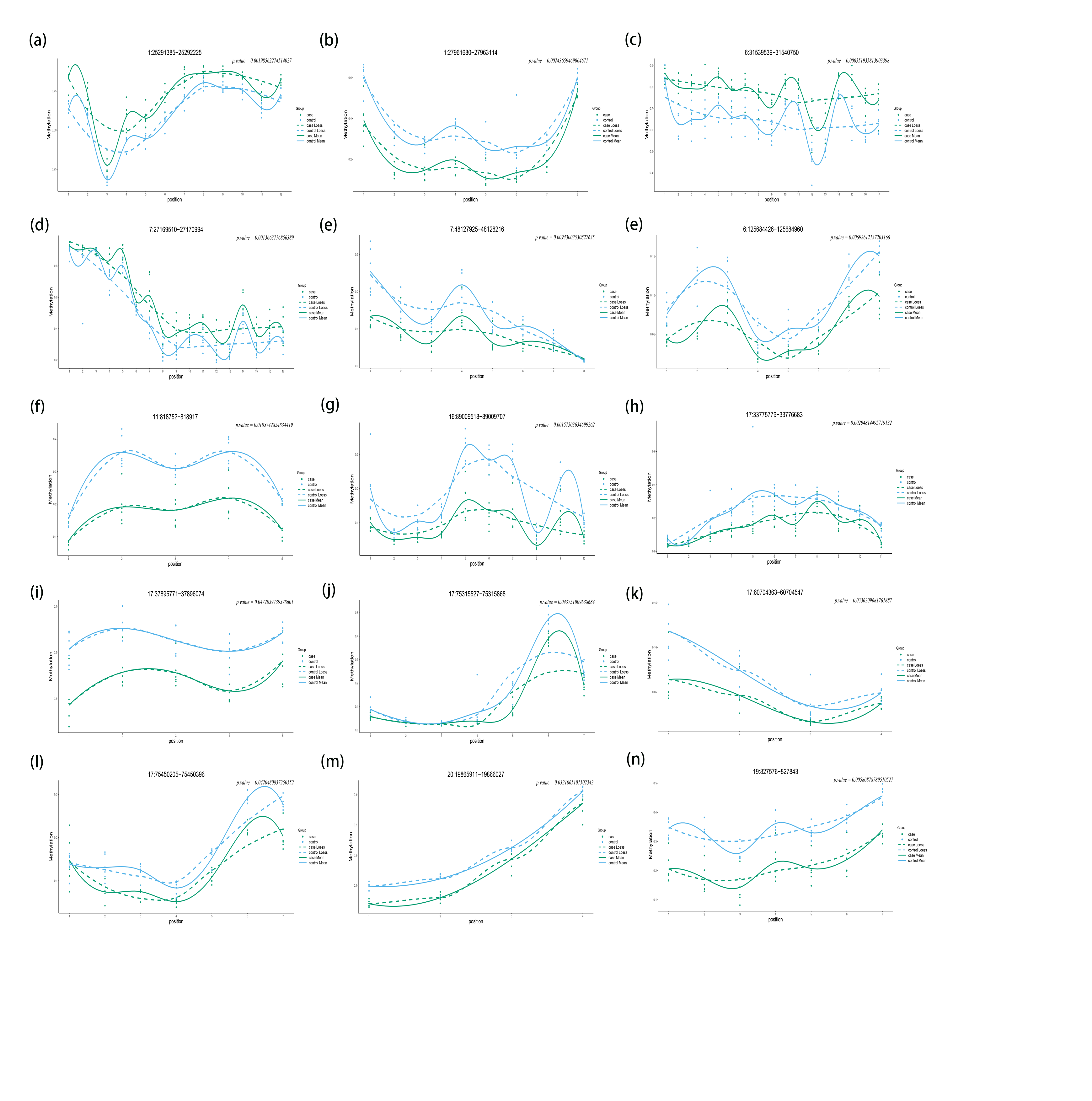

Supplement: Supplementary file 1 [file antioxidants-14-00835-s001.zip › Supplementary figures 1-6/supplemental figure 2.tif]

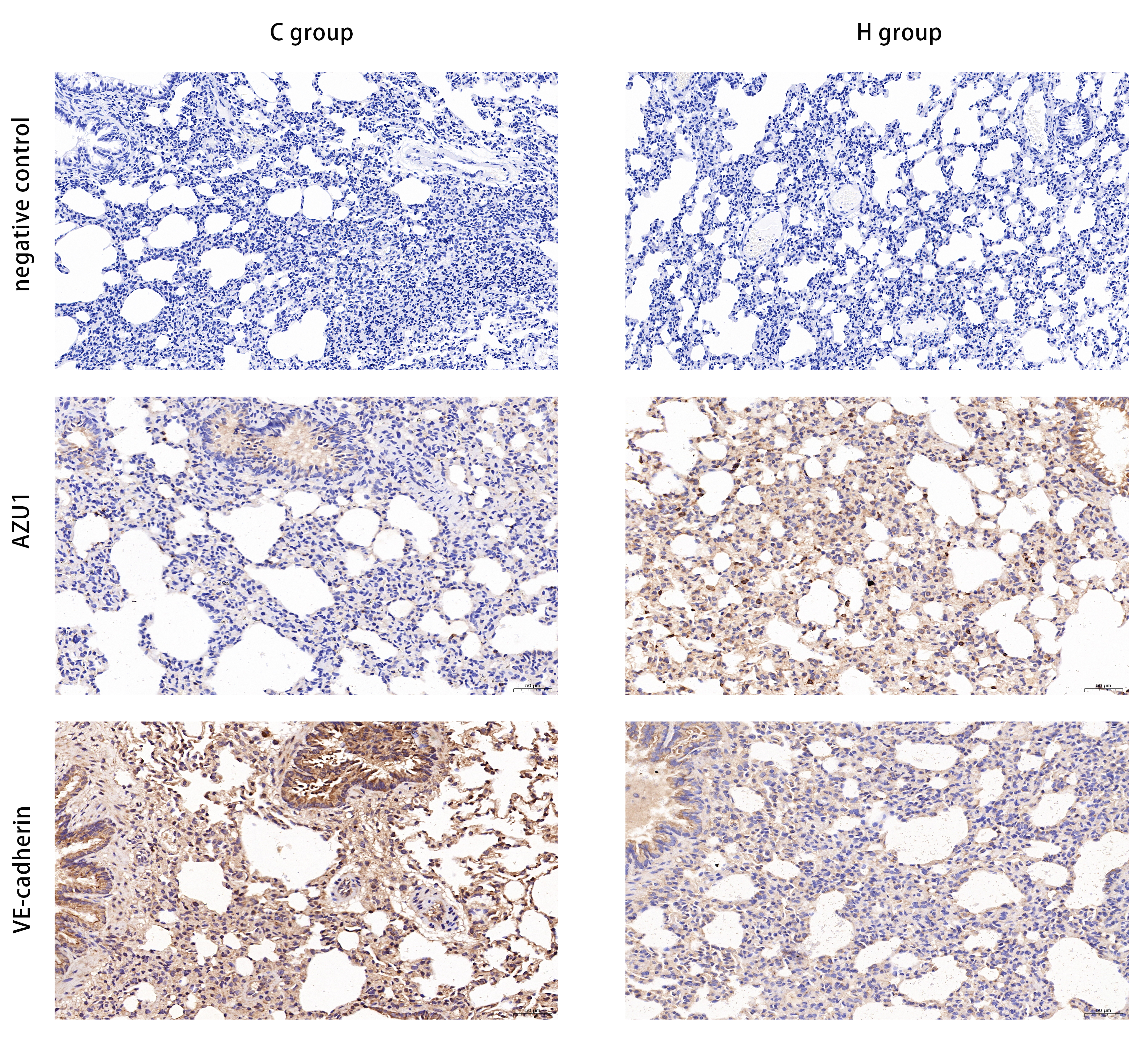

Supplement: Supplementary file 1 [file antioxidants-14-00835-s001.zip › Supplementary figures 1-6/supplemental figure 3.tif]

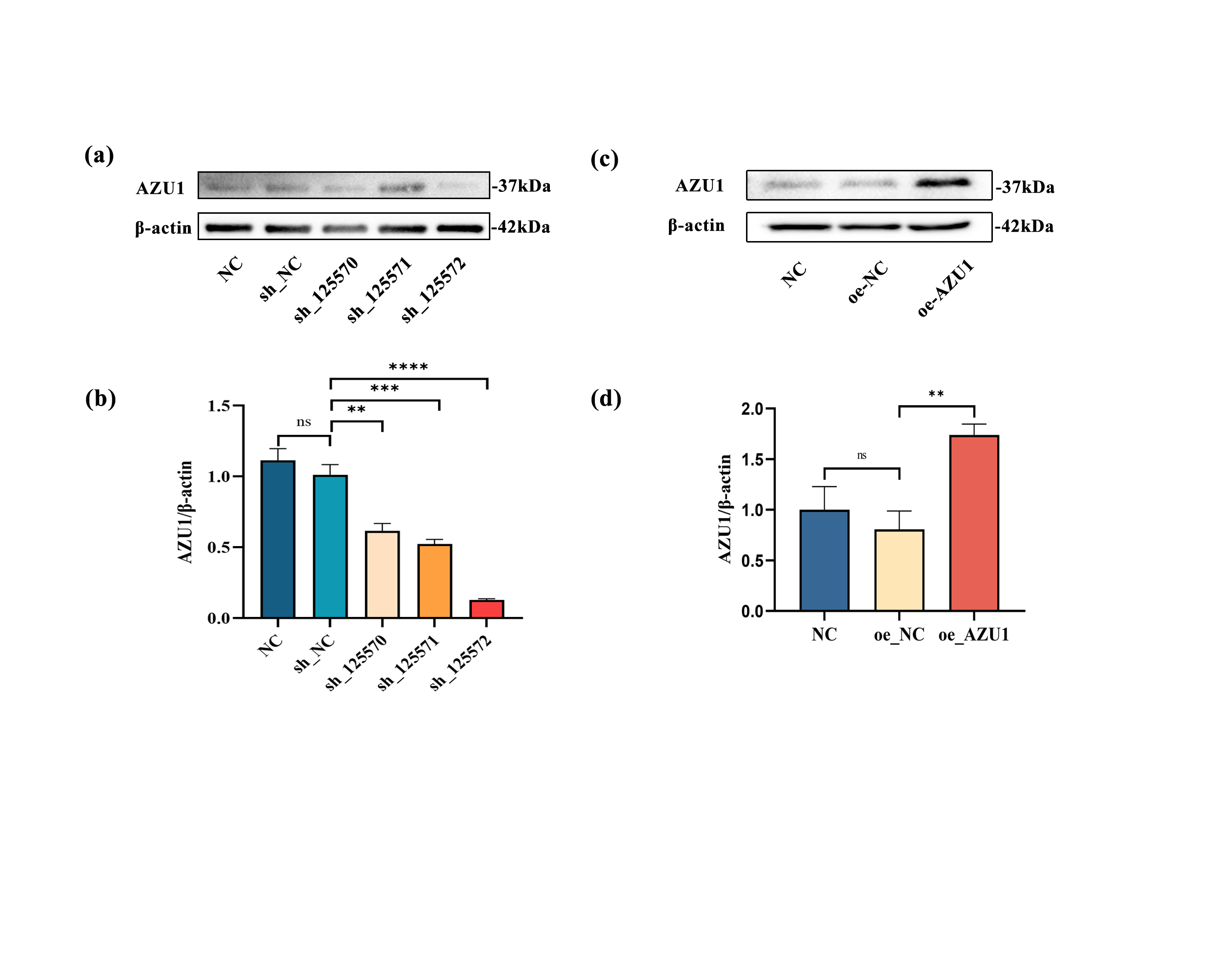

Supplement: Supplementary file 1 [file antioxidants-14-00835-s001.zip › Supplementary figures 1-6/supplemental figure 4.tif]

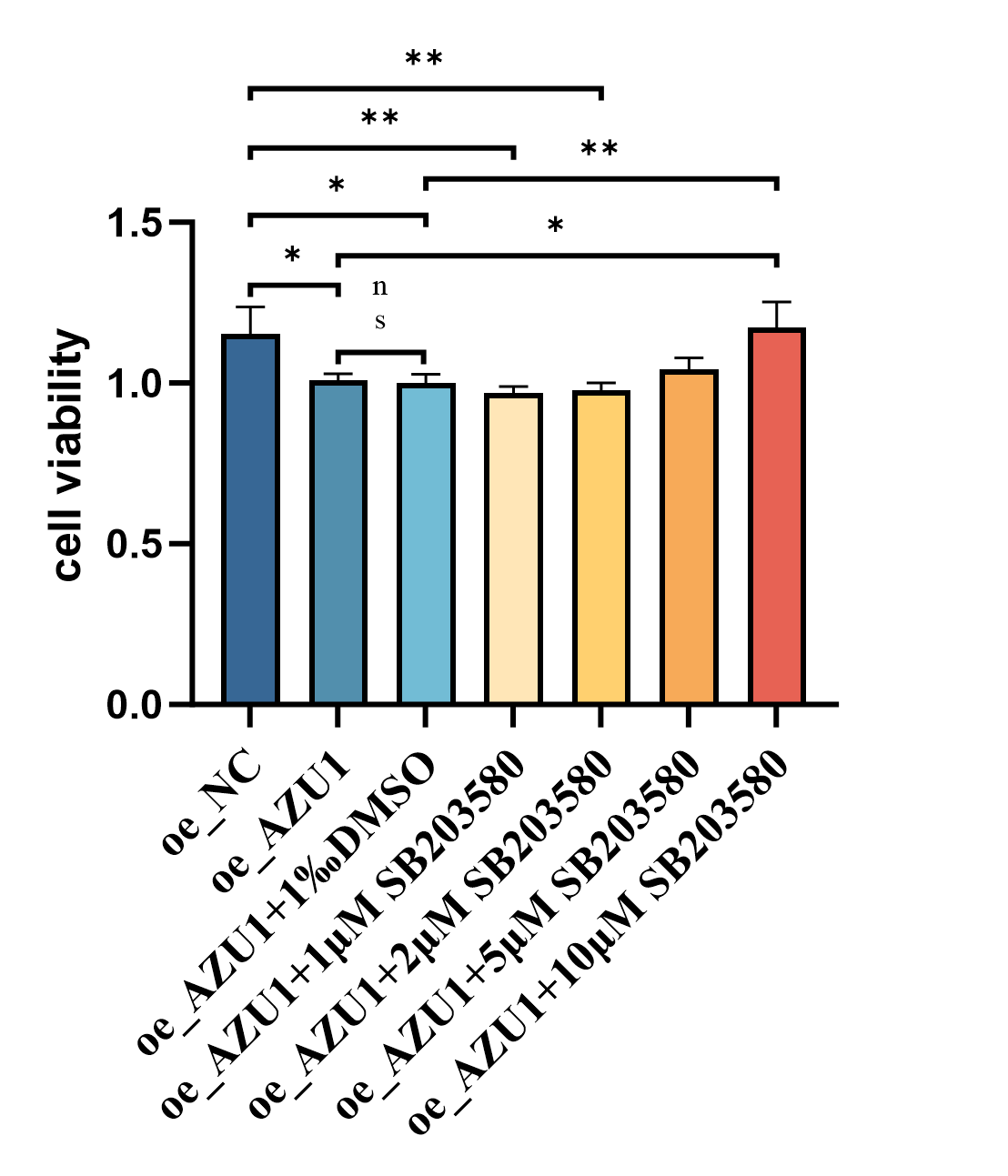

Supplement: Supplementary file 1 [file antioxidants-14-00835-s001.zip › Supplementary figures 1-6/supplemental figure 5.tif]

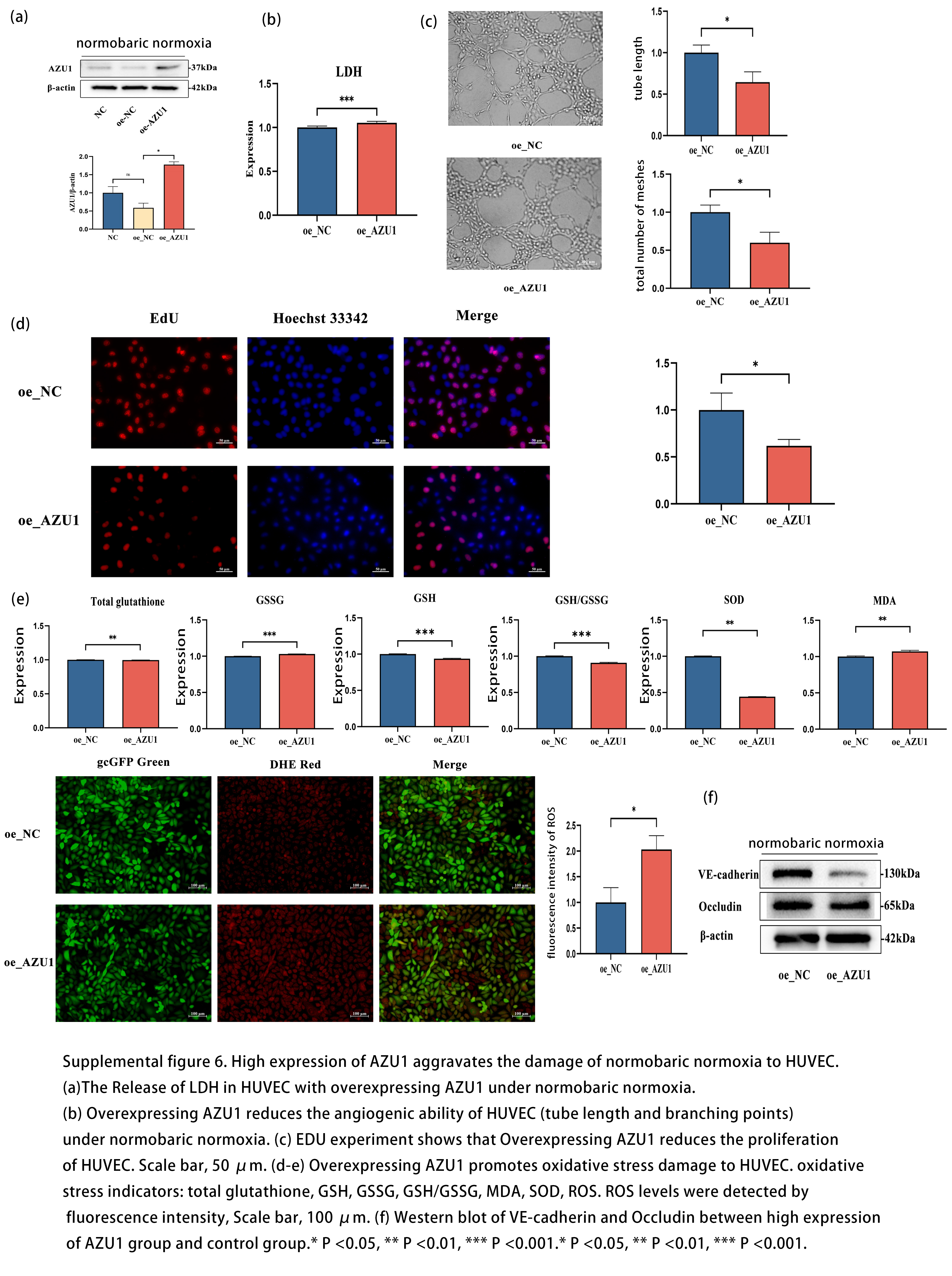

Supplement: Supplementary file 1 [file antioxidants-14-00835-s001.zip › Supplementary figures 1-6/supplemental figure 6.tif]
